# Construction of a miRNA Panel for Differentiating Lung Adenocarcinoma Brain Metastases and Glioblastoma

**DOI:** 10.3390/cancers17040581

**Published:** 2025-02-08

**Authors:** Bernadett Torner, Dóra Géczi, Álmos Klekner, István Balogh, András Penyige, Zsuzsanna Birkó

**Affiliations:** 1 Department of Human Genetics, Faculty of Medicine, University of Debrecen, 4032 Debrecen, Hungary; torner.bernadett@med.unideb.hu (B.T.); g.dora@med.unideb.hu (D.G.); balogh@med.unideb.hu (I.B.); penyige@med.unideb.hu (A.P.); 2 Department of Neurosurgery, Faculty of Medicine, University of Debrecen, 4032 Debrecen, Hungary; klekner.almos@med.unideb.hu; 3 Division of Clinical Genetics, Department of Laboratory Medicine, University of Debrecen Clinical Center, 4032 Debrecen, Hungary

**Keywords:** lung adenocarcinoma brain metastasis, glioblastoma, miRNAs, next-generation sequencing, brain tissue, biomarker panel

## Abstract

Background/Objectives: Brain metastases (BM) are the most common type of intracranial malignant tumor and are associated with high mortality. More than 50% of BM cases originate from lung cancer, and lung adenocarcinoma (LUAD) is most commonly associated with the development of BM (25%). The differential diagnosis of solitary BM and glioblastoma (GBM)—one of the most aggressive and fatal malignant brain tumors—remains a considerable challenge. Given the major role of microRNAs (miRNAs) in regulating gene expression, their clinical potential as biomarkers for tumor diagnosis and prognosis offers significant promise. Methods: Next-generation RNA Sequencing (RNA-seq) was used to assess the miRNA expression profiles of 6 LUAD-BM, 6 GBM, and 6 control (non-tumoral brain tissue samples) human brain tissue samples. miRNAs exhibiting the most significant differential expression in LUAD-BM patients in comparison to both control subjects and GBM patients were selected for validation through RT-qPCR. Results: The analysis of RNA-seq data revealed the presence of 229 differentially expressed miRNAs in the comparison between LUAD-BM and control samples and 46 in the comparison between LU-AD-BM and GBM samples. Eight miRNAs were selected for further analysis, four of which were upregulated and four downregulated, based on the significant differences in their expression levels observed between the LUAD-BM samples and the other two groups, as confirmed with the Mann–Whitney U test. Functional enrichment analysis was also conducted based on a miRNA-centered target analysis performed using the miRNet tool. To assess the diagnostic potential of these differentially expressed miRNAs, we performed a receiver operating characteristic (ROC) curve analysis. Conclusions: A panel of eight miRNAs was identified in human brain tissue samples, exhibiting high accuracy in distinguishing LUAD-BM from both GBM and control samples.

## Simple Summary

This study addresses the challenge of differentiating lung adenocarcinoma brain metastases (LUAD-BM), the most common malignant lesion of the central nervous system (CNS), from glioblastoma (GBM), a grade IV glioma. Both tumors exhibit similar features on conventional MRI, limiting its diagnostic accuracy. Our goal was to explore the potential of microRNAs (miRNAs), key regulators of gene expression, as biomarkers for LUAD-BM. Using next-generation RNA sequencing, we analyzed miRNA profiles from LUAD-BM, GBM, and control brain tissue samples. We identified eight miRNAs with significantly different expression in LUAD-BM compared to GBM and control tissues. These miRNAs demonstrated high accuracy in distinguishing LUAD-BM from the other two groups.

## 1. Introduction

Brain metastases (BM) are regarded as the most prevalent malignant lesions of the central nervous system (CNS), with 40–50% of BM originating from lung cancer. Lung adenocarcinoma (LUAD), the most prevalent pathological type of non-small-cell lung cancer (NSCLC), is correlated with the development of BM in 25% of cases [1,2,3]. The reported incidence of BM shows an increasing trend, which can be the consequence of improved therapeutic options, leading to increased survival among patients diagnosed with primary tumors, as well as the wider adoption of advanced imaging diagnostic technologies, which have enabled the early identification of BM [4]. The prognosis for patients with BM is extremely unfavorable, and the currently available therapeutic options are also unsatisfactory. The average survival time of untreated lung cancer patients with BM is only 1–3 months, which can be extended to 12 months with treatment [5,6]. Current routine diagnosis includes computed tomography (CT) or magnetic resonance imaging (MRI); however, approximately 30% of multiple metastases cannot be identified reliably, even with gadolinium-enhanced contrast material [7,8]. Therefore, the identification of alternative biomarkers to facilitate the diagnosis and differentiation of BM from other tumors is warranted.

Glioblastoma (GBM) is a malignant, diffusely growing primary tumor affecting the CNS. According to the World Health Organization (WHO), it can be classified as a grade IV glioma. GBM accounts for approximately 50% of primary CNS tumors, with an incidence of 10 cases per 100,000 people [9]. GBM primarily affects individuals over the age of 45, with male predominance. Due to the aggressive nature of the disease, the median overall survival time from diagnosis is estimated to be 15 months, while the 5-year survival rate is ~5% [9,10].

Differentiating between unknown malignant primary brain tumors and solitary BMs using conventional MRI remains challenging due to overlapping imaging characteristics. Consequently, histological confirmation through biopsy is often required to establish the diagnosis [11,12]. It is well established that, among primary brain tumors, the MRI pattern of GBM is very similar to that of BM; consequently, the distinction between these entities represents a substantial challenge for neuroradiologists. Both tumor types exhibit similar intratumoral texture and peritumoral hyperintensity on the basis of conventional MRI examination. In the case of BMs, peritumoral hyperintensity observed on imaging arises from an increase in interstitial water levels, driven by capillary permeability and breakdown of the blood–brain barrier. GBMs are surrounded by infiltrative edema, characterized by the presence of infiltrating tumor cells in addition to interstitial water [13]. BMs are characterized by early spread in many cases, frequently appearing synchronously with the primary tumor; therefore, it can be challenging to determine the origin of the primary cancer, further complicating the diagnostic process [14]. The accurate diagnosis is extremely important, as the treatment of the two tumor types differs markedly [15,16]. GBM patients typically receive radiotherapy and temozolomide chemotherapy after total surgical resection [17]. BM treatment strategies depend on the size, location, and type of primary tumor, typically combining stereotactic radiosurgery, surgical resection, and whole-brain radiation therapy. Systemic chemotherapy is not routinely used for BMs, as they often respond more effectively to targeted primary anticancer therapies [18]. In cases of epidermal growth factor receptor (EGFR) mutant BMs from NSCLC, targeted agents such as gefitinib and erlotinib are effective treatment options [19,20]. Numerous studies have focused on identifying BM biomarkers of lung cancer origin, mostly focusing on clinical factors or molecular biomarkers, but significant development has not been achieved yet in those areas.

Current biomedical research is increasingly focusing on the physiological and pathophysiological role of epigenetic regulatory mechanisms. Non-coding RNAs, including microRNAs (miRNAs), are receiving increasing attention. These molecules are considered promising biomarker candidates because of their crucial involvement in regulating pathogenic and tumorigenic processes [21]. miRNAs are small, evolutionarily conserved, non-coding RNAs, approximately 21–25 nucleotides in length, which can affect more than 60% of the human genome protein-coding genes. It is known that miRNAs bind to the 3′-untranslated region (3′-UTR) of specific target mRNAs, thereby inducing either target mRNA degradation or translational repression, which ultimately results in gene silencing [22,23]. These non-coding RNA molecules play a crucial role in the regulation of fundamental biological processes, such as cell proliferation, cell differentiation, apoptosis, migration, and cell metabolism. Their deregulation is implicated in various cancer-related processes [24,25]. The clinical potential of miRNAs in tumor diagnosis and prognosis is currently under extensive investigation, as they can function both as oncogenes (by silencing tumor suppressor mRNAs) or tumor suppressors (by down-regulating oncogenic miRNAs). Mapping aberrant miRNA expression has significant diagnostic and prognostic potential [26]. Due to limited treatment options, late diagnosis, and difficulties in identifying BM, the odds for patients remain extremely poor. It is crucially important to identify new biomarkers that can reliably distinguish between different types of brain tumors, potentially improving patients’ survival rates.

In the present study, we aimed to map the miRNA profile using intraoperative tissue samples of LUAD-BM and GBM patients treated at the Department of Neurosurgery of the Faculty of Medicine, University of Debrecen. Our goal is to identify miRNAs whose expression change can be associated with metastatic processes while at the same time helping to identify and differentiate BM from other types of brain tumors, as well as to understand the differences in invasion properties. For this purpose, we identified miRNAs with significantly different expression patterns in LUAD and GBM tissue samples using high-throughput next-generation sequencing (NGS) and validated the results with reverse transcription-quantitative polymerase chain reaction (RT-qPCR). Additionally, Gene Ontology (GO) and Kyoto Encyclopedia of Genes and Genomes (KEGG) pathway analyses were performed to assess the significance of these miRNAs in tumorigenesis and to map invasion differences.

## 2. Materials and Methods

### 2.1. Sample Collection

This study included patients diagnosed with LUAD-BM and GBM who were treated at the Neurosurgical Department of the Faculty of General Medicine of the University of Debrecen. The study groups consisted of patients diagnosed with GBM and LUAD-BM, while the control group included peritumoral brain tissue samples from patients with low-grade glioma (grade I, II) who underwent supramaximal resection as part of a neurosurgical tumor removal procedure. For NGS analysis, 6 GBM, 6 LUAD-BM, and 6 control samples were selected, with an additional 30 patients from each study group included in the validation process. The intraoperative tissue samples of patients were confirmed by histopathological examination, and none of them received chemotherapy or radiation therapy. The average age of LUAD-BM patients was 61.46 years, while that of GBM patients was 59.33 years; the controls were selected to closely match the patients in age (Table 1). The gender ratio was consistent across all three study groups, with equal proportions of male and female participants. After surgery, the flash-frozen tissue samples were stored at −80 °C until use. The Scientific and Research Ethics Committee of the Medical Research Council of the Ministry of Health, Budapest, Hungary (ETT TUKEB; project identification code: 51450/2015/EKU (0411/15)), approved the study, which was consistent with the Declaration of Helsinki, and all participants signed a consent form.

### 2.2. Tissue Sample Disruption and RNA Extraction

For total RNA purification, 30 mg/sample of flash-frozen tissue samples were dissected on ice. The tissue samples were disrupted and homogenized with a MagNa Lyser device (Roche Ltd., Basel, Switzerland), using Qiazol lysis reagent and stainless steel beads. MiRNA-enriched total RNA isolation was performed according to the manufacturer’s instructions using the miRNeasy Mini Kit (Qiagen, Hilden, Germany). The quantity and quality of the isolated RNA were determined using a Nanodrop spectrophotometer (Thermo Scientific, Waltham, MA, USA).

### 2.3. Library Preparation and Next-Generation Sequencing (NGS)

To compare miRNA expression patterns among LUAD-BM, control, and GBM tissue samples, NGS was performed on 18 selected brain tissue samples in collaboration with the Genomic Medicine and Bioinformatics Core Facility (Department of Biochemistry and Molecular Biology, Faculty of Medicine, University of Debrecen), using the Illumina NextSeq500 (Illumina, San Diego, CA, USA) platform. The NEBNext Multiplex Small RNA Perp Set for Illumina (1-48) 96 rxn kit (New England BioLabs, Ipswich, MA, USA) was used for library preparation, for which the quality of the RNA samples was examined with the Eukaryotic Total RNA Nano Assay on the Agilent BioAnalyzer (Agilent Technologies, Santa Clara, CA, USA). For a small RNA-seq library, 1 µg of total RNA with an RNA integrity number (RIN) score of >7 was used. Fragment size distribution and molarity of libraries were also checked on the Agilent BioAnalyzer DNA1000 chip (Agilent Technologies, Santa Clara, CA, USA). Finally, the libraries were sequenced on an Illumina NextSeq 500 Sequencing System (Illumina, San Diego, CA, USA) with read lengths of 50 base pairs (single reads). Fastq files were aligned to the human reference genome (GRCh38) using the Novoalign algorithm. Downstream analysis of the generated BAM files was performed with the StrandNGS v4.0 software (www.strand-ngs.com (accessed on 3 March 2021) and normalization was carried out with the DESeq algorithm. A moderated t-test was used to identify differentially expressed miRNAs.

### 2.4. Identification of Differentially Expressed miRNAs

Normalized expression values of miRNAs were analyzed using the iDEP 1.1 tool (http://bioinformatics.sdstate.edu/idep11; (accessed on 25 April 2023)). Within iDEP, hierarchical clustering was conducted with a Z score cutoff of 3, followed by K-Means clustering of the 100 most variable miRNAs and principal component analysis (PCA). Differential gene expression was assessed using the DESeq2 software package (iDEP 1.1 software); the DEG1 analysis was performed with a false discovery rate (FDR) cutoff of 0.1 and a minimum fold change (FC) of 2. Significantly upregulated miRNAs were defined with an FC of ≥2 and an FDR of ≤0.05, while miRNAs with an FC of ≤−2 and an FDR of ≤0.05 were classified as downregulated.

### 2.5. Functional Annotation and Enrichment Analysis

To predict experimentally validated targets regulated by differentially expressed miRNAs, we used the miRNet tool (https://www.mirnet.ca (accessed on 27 June 2024)) with the miRTarBase v9.0 database (https://awi.cuhk.edu.cn/~miRTarBase/miRTarBase_2025/php/index.php (accessed on 15 September 2021)) to create an interaction network. During the analysis, we applied the minimum network option and conducted GO analysis—covering biological process (BP)—as well as KEGG pathway enrichment analysis, using the KEGG and GO BP databases within miRNet. Enrichment analysis for GO and KEGG pathways was considered statistically significant at *p* < 0.05. Common target genes among experimentally validated miRNAs were identified using the miRTarBase v9.0 database.

### 2.6. Verification of miRNA Expression Levels Through Quantitative Real-Time PCR (RT-qPCR)

For the validation process, we performed a reverse transcription on total RNA samples from 30 control subjects, 30 LUAD-BM, and 30 GBM patients using the miRCURY LNA RT Kit (Qiagen, Hilden, Germany) under conditions of 42 °C for 60 min and 95 °C for 5 min. To determine the expression level of has-miR-200c-5p, hsa-miR-141-5p, hsa-miR-375-3p, hsa-miR-383-5p, hsa-miR-200a-5p, hsa-miR-129-2-3p, hsa-miR-410-3p, and hsa-miR-9-5p, quantitative real-time PCR reaction was performed using the LightCycler^®^ 96 instrument (Roche Ltd., Pleasanton, CA, USA) and the miRCURY LNA SYBR Green PCR Kit (Qiagen, Hilden, Germany) according to the manufacturer’s instructions. PCR reaction conditions were set as follows: initial denaturation at 95 °C for 120 s, followed by 45 amplification cycles (denaturation at 95 °C for 10 s; annealing and elongation at 56 °C for 60 s). A 3-step melting curve analysis was then performed (95 °C for 20 s, 40 °C for 20 s, 85 °C for 1 s), followed by a final cooling step at 37 °C for 30 s. The expression level of the tested miRNAs was determined using the comparative cycle threshold (∆∆Ct) method; the miRNA expression values were normalized to hsa-miR-103a-3p expression (∆Ct = Ct target miRNA—Ct hsa-miR-103a-3p) [27]. Experiments were performed in triplicates.

### 2.7. Statistical Analysis

Statistical significance was analyzed by the non-parametric Mann–Whitney U test using the GraphPad Prism 7 program; miRNA expression differences were considered significant at *p* < 0.05. The receiver operating characteristic (ROC) curve analysis was conducted using the easyROC 1.3.1 software (http://biosoft.erciyes.edu.tr/app/easyROC/ (accessed on 25 July 2016)). The area under the curve (AUC) value was obtained from the ROC analysis, with the optimal cut-off value determined depending on the sensitivity and specificity values. The diagnostic efficiency of the validated miRNA panel was assessed using IBM SPSS Statistics 27. A binary logistic regression model was applied, followed by ROC analysis based on the calculated probability values.

## 3. Results

### 3.1. Hierarchical Clustering, K-Means Clustering, and PCA of Normalized Mirna-Seq Data

**Figure 1:**
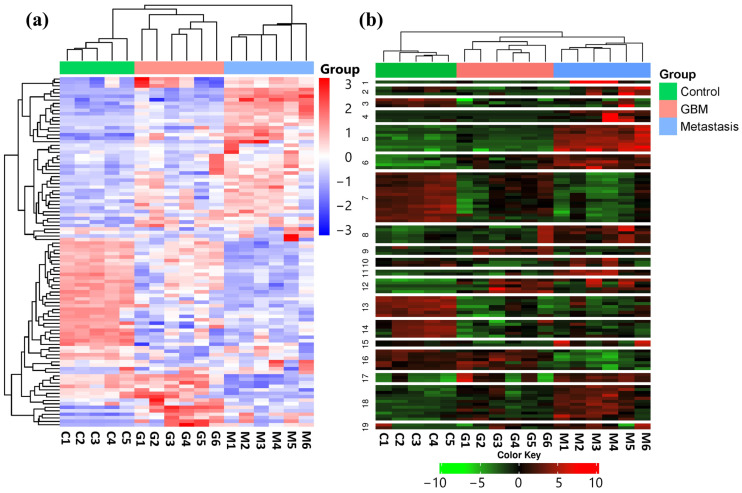
(**a**) Hierarchical cluster analysis of the 100 most variable miRNAs in the LUAD-BM, GBM, and control groups is visualized on the heatmap. Columns represent individual samples for the three study groups, while rows display the expression patterns of individual miRNAs, represented with a color scale. Upregulated miRNAs are shown in blue, while downregulated miRNAs are shown in red. (**b**) The heatmap illustrates the results of K-Means clustering for the top 100 differentially expressed miRNAs. (Color code: control—green; GBM—red; metastasis—blue.)

In order to identify expression differences between the LUAD-BM, GBM, and control groups, a hierarchical cluster analysis was conducted using normalized NGS expression data. The analysis included samples for each group. Based on standard deviation, the top 100 miRNAs with the largest expression differences were selected for investigation. The heatmap illustrates the expression differences and similarities of the samples belonging to the aforementioned three groups, as illustrated in Figure 1a.

Using K-Means clustering, miRNAs were grouped into 19 clusters based on the expression patterns of all analyzed samples (Figure 1b). The clustering was again performed on the top 100 miRNAs having the most distinct expression patterns, ranked by standard deviation.

**Figure 2:**
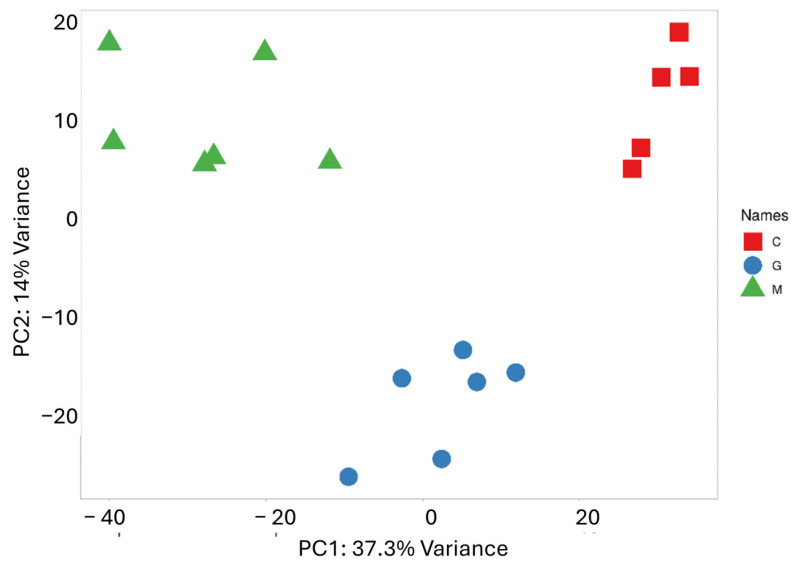
PCA based on the miRNA expression profile shows that LUAD-BM patients (green) are well separated from controls (red) along PC1 and from GBM patients (blue) along PC2.

PCA was performed to reduce the dimensionality of the data and capture the overall distribution of miRNA expression values (Figure 2). Along the PC1 axis, which explains the largest variance (37.3%), a clearly distinct expression pattern between the metastatic group and the control group is observed. Meanwhile, the difference between the metastatic and GBM groups is less pronounced, though some separation is evident along the PC2 axis, which accounts for 14.1% of the variance. As expected, the scatter plot shows samples that belong to the same group are closely clustered together. The results of the cluster analysis and PCA clearly confirm the distinct miRNA expression profile of the two tumor types, which correlate with their pathological characteristics.

### 3.2. miRNA Differential Expression Analysis

Using the DESeq2 algorithm, we identified significantly differentially expressed miRNAs in the LUAD-BM–control and LUAD-BM–GBM comparison. The screening was performed with a false discovery rate (FDR) of <0.1 and a fold change of >2. In the case of the LUAD-BM–control comparison, 111 downregulated and 118 upregulated miRNAs were identified, while in the LUAD-BM–GBM comparison, 16 downregulated and 30 upregulated miRNAs were found (Tables S1 and S2).

**Figure 3:**
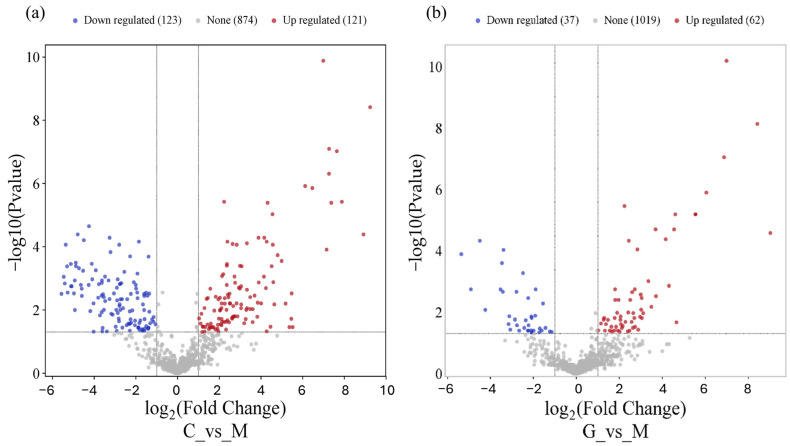
The volcano plot shows that LUAD-BM (**a**) and GBM (**b**) development leads to a remarkable change in the miRNA transcriptome.

As shown in Figure 3, the common miRNAs among the top 15 upregulated miRNAs in both comparisons were hsa-miR-200c-3p (logFC = 9.24, logFC = 8.42), hsa-miR-375-3P (logFC = 8.92, logFC = 9.03), hsa-miR-21-3P (logFC = 7.88, logFC = 3.49), hsa-miR-200A-5P (logFC = 7.64, logFC = 6.05), hsa-miR-210-3P (logFC = 7.37, logFC = 3.7), hsa-miR-141-3P (logFC = 7.28, logFC = 6.87), hsa-miR-200A-3P (logFC = 7.26, logFC = 5.54), hsa-miR-141-5P (logFC = 6.99, logFC = 6.99), hsa-miR-200B-3P (logFC = 6.46, logFC = 5.54), and hsa-miR-429 (logFC = 6.12, logFC = 4.54). Among the top 15 most downregulated miRNAs, the shared miRNAs were hsa-miR-135a-5p (logFC = −5.67, logFC = −5.70) and hsa-miR-9-3p (logFC = −5.2, logFC = −5.63). The volcano plot visualizes differentially expressed miRNAs in the LUAD-BM–control comparison (Figure 3a) and LUAD-BM–GBM comparison (Figure 3b).

### 3.3. Network-Based Analysis of Differentially Expressed miRNAs

**Figure 4:**
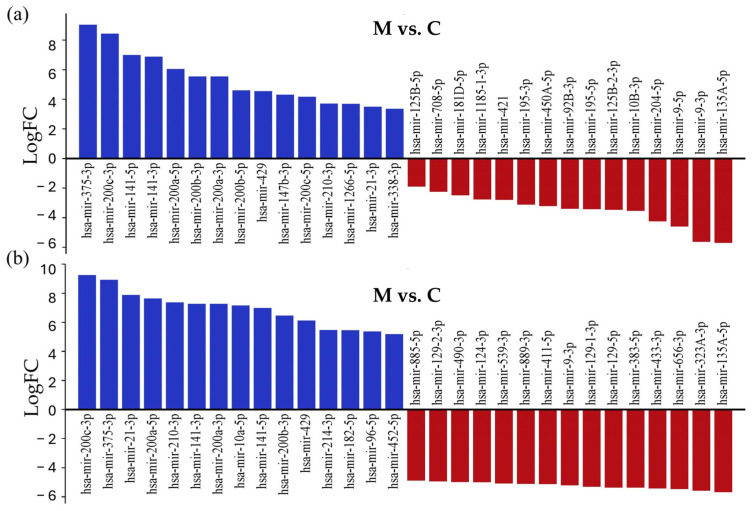
List of the 15 most strongly downregulated and upregulated miRNAs in tissue samples of LUAD-BM patients, compared with their expression in the control group (**a**) and GBM patients (**b**), ranked according to their expression levels.

To identify experimentally validated miRNA targets and identify miRNA functions, we conducted miRNA-centric network analysis using the miRNet 2.0 online tool. During the analysis, the 15 most upregulated and downregulated miRNAs with the largest differences in log2FC values were examined in both comparisons (Figure 4); the whole list is presented in Table S3.

**Figure 5:**
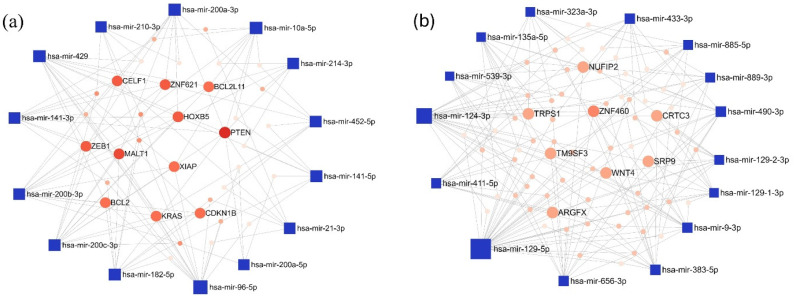
The minimum interaction network of (**a**) upregulated and (**b**) downregulated miRNAs in the LUAD-BM vs. control comparison, along with their experimentally validated target genes, was generated using the miRNet tool. In the network, blue squares represent miRNAs, while red circles denote target genes. Node size is proportional to the degree value, and color intensity reflects the betweenness centrality.

In the LUAD-BM–control comparison, the top 15 upregulated miRNAs with the highest degree values were hsa-miR-96-5p (degree 15), hsa-miR-200c-3p (degree 13), and hsa-miR-429 (degree 13). Among the target genes, Phosphatase And Tensin Homolog (PTEN; degree 9) and MALT1 Paracaspase (MALT1; degree 7) had the highest number of interacting partners. For the downregulated miRNAs, hsa-miR-129-5p (degree 34), hsa-miR-124-3p (degree 25), and hsa-miR-656-3p (degree 13) showed the highest degree values, while Zinc Finger Protein 460 (ZNF460; degree 6) emerged as the most prominent target gene. The miRNA–target interaction networks generated by miRNet for the upregulated and downregulated miRNAs are illustrated in Figure 5.

**Figure 6:**
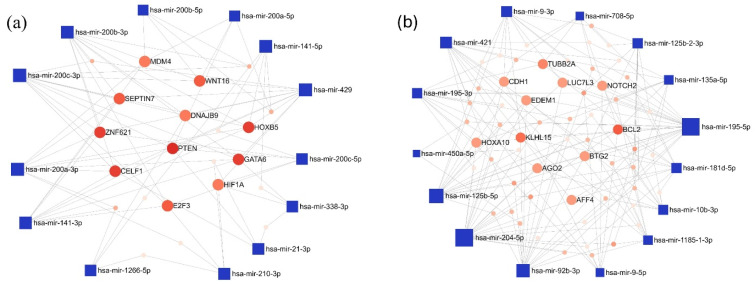
The minimum network of the (**a**) upregulated and (**b**) downregulated miRNAs in the LUAD-BM—GBM comparison and their experimentally validated target genes generated by the miRNet tool. (Blue square—miRNAs; red spots—target genes; size—degree value; color intensity—betweenness value).

Furthermore, the interaction network of the miRNAs exhibiting the most pronounced expression differences in the LUAD-BM-GBM comparison was also mapped. Among the top 15 upregulated miRNAs, the highest number of connections were observed for hsa-miR-200c-3p (degree 11), hsa-miR-200b-3p (degree 10), and hsa-miR-429 (degree 10). For target genes, PTEN had the highest degree value (degree 9), followed by a few target genes with a common degree value of 6: GATA Binding Protein 6 (GATA), Homeobox B5 (HOXB5), CUGBP Elav-like Family Member 1 (CELF1), and Zinc Finger Protein 621 (ZNF621) (Figure 6a). Among the downregulated miRNAs, hsa-miR-204-5p (degree 22), hsa-miR-195-5p (degree 22), and hsa-miR-92b-3p (degree 17) showed the highest interactions, while BCL2 Apoptosis Regulator (BCL2; degree 7) and Kelch-like Family Member 15 (KLHL15; degree 6) were the most connected target genes (Figure 6b).

### 3.4. Gene Ontology (GO) and Pathway Enrichment Analysis of MiRNA

**Figure 7:**
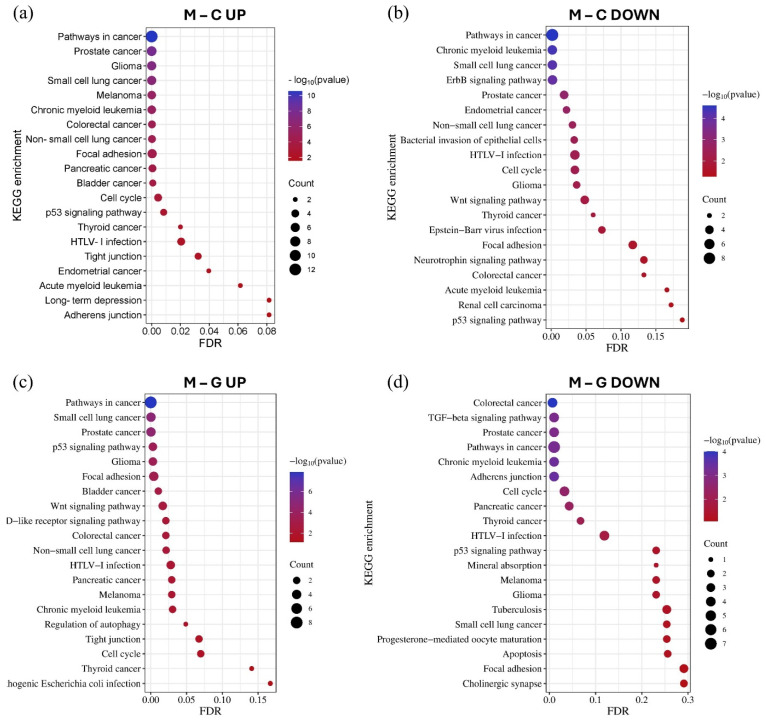
The KEGG pathway enrichment analysis of the top 15 upregulated (**a**) and downregulated (**b**) miRNAs in the LUAD-BM–control comparison, as well as the top 15 upregulated (**c**) and downregulated (**d**) miRNAs in the LUAD-BM–GBM comparison based on the results of the miRNet tool. The size of the dots represents the number of genes involved in the given KEGG pathway, while their significance is characterized by their FDR and −log10 *p* values.

Using the GO and KEGG databases accessed through the miRNet tool, we performed miRNA–target gene network-based functional enrichment and pathway analysis. This allowed us to independently evaluate the enrichment results for miRNAs exhibiting the most significant expression differences in the LUAD-BM–control and LUAD-BM–GBM comparisons. The analysis was performed based on the interaction networks of the top 15 upregulated and top 15 downregulated miRNAs identified in each comparison. KEGG pathway analysis revealed that miRNAs upregulated in LUAD-BM compared to controls predominantly regulate key processes, including the p53 signaling pathway, cell cycle, focal adhesion, tight junctions, and adherens junctions. In contrast, downregulated miRNAs were enriched in pathways such as the ErbB signaling pathway, focal adhesion-related pathways, and the Wnt signaling pathway. In the LUAD-BM to GBM comparison, upregulated miRNAs showed enrichment in the p53 signaling pathway, cell cycle regulation, tight junctions, focal adhesion, and autophagy regulation, among others. Downregulated miRNAs were associated with the transforming growth factor beta (TGF-beta) signaling pathway, cell cycle regulation, adherens junctions, and focal adhesion. Notably, the deregulation of these miRNAs was consistently linked to enrichment in NSCLC pathways (Figure 7).

**Figure 8:**
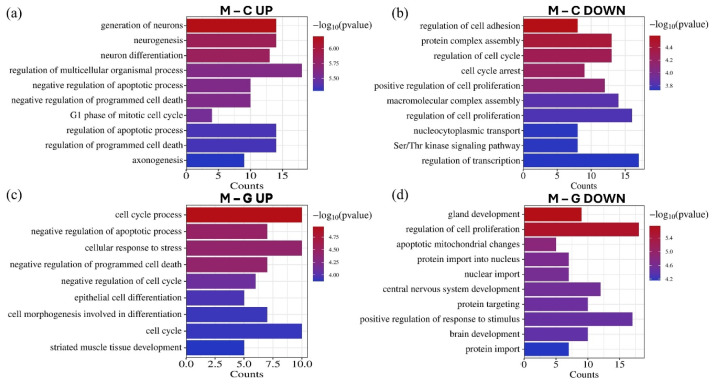
The GO biological process enrichment annotation of the top 15 upregulated (**a**) and downregulated (**b**) miRNAs in the LUAD-BM–control comparison, as well as the top 15 upregulated (**c**) and downregulated (**d**) miRNAs in the LUAD-BM–GBM comparison based on the results of the miRNet tool. The significance of the identified biological processes is represented by their −log10 *p*-values.

GO biological process enrichment analysis revealed that the significantly differentially expressed miRNAs regulate genes implicated in various tumor-related processes (Figure 8). Upregulated miRNAs were associated with the negative regulation of apoptosis, negative regulation of programmed cell death, and cell cycle, while downregulated miRNAs were linked to the positive regulation of cell proliferation. Furthermore, these miRNAs influence processes related to invasion, such as the regulation of cell adhesion. Additionally, several enriched processes were identified in CNS development, including neurogenesis, neuron differentiation, axonogenesis, and brain development.

### 3.5. Validation of Differentially Expressed miRNAs by RT-qPCR in Tissue Samples

**Figure 9:**
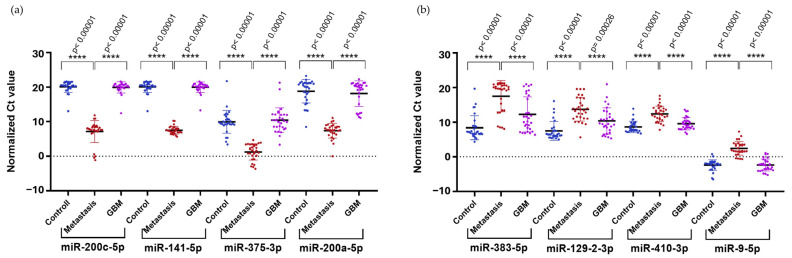
Significance analysis of differentially expressed miRNAs. (**a**) Upregulated miRNAs and (**b**) downregulated miRNAs were evaluated using the Mann–Whitney U test. (Color code: LUAD-BM—red; control—blue; GBM—magenta; **** *p* < 0.0001).

To confirm the results obtained from NGS in a larger cohort, we selected the upregulated hsa-miR-200c-5p (logFC = 4.16), hsa-miR-141-5p (logFC = 6.99), hsa-miR-200a-5p (logFC = 7.64), and hsa-miR-375-3p (logFC = 8.92), along with the downregulated hsa-miR-410-3p (logFC = −4.73) and hsa-miR-9-5p (logFC = −4.64) identified in the comparison of LUAD-BM and GBM for RT-qPCR validation. We selected two additional miRNAs, hsa-miR-383-5p (logFC = −5.36) and hsa-miR-129-2-3p (logFC = −4.93), from the LUAD-BM–control comparison. Normalization was performed using hsa-miR-103a-3p, and all measurements were carried out in triplicates. RT-qPCR results were analyzed using the Mann–Whitney U test, which confirmed significant upregulation of hsa-miR-200c-5p, hsa-miR-141-5p, hsa-miR-200a-5p, and hsa-miR-375-3p (Figure 9a) and significant downregulation of hsa-miR-383-5p, hsa-miR-129-2-3p, hsa-miR-410-3p, and hsa-miR-9-5p (Figure 9b) in LUAD-BM samples compared to both GBM and control samples.

**Figure 10:**
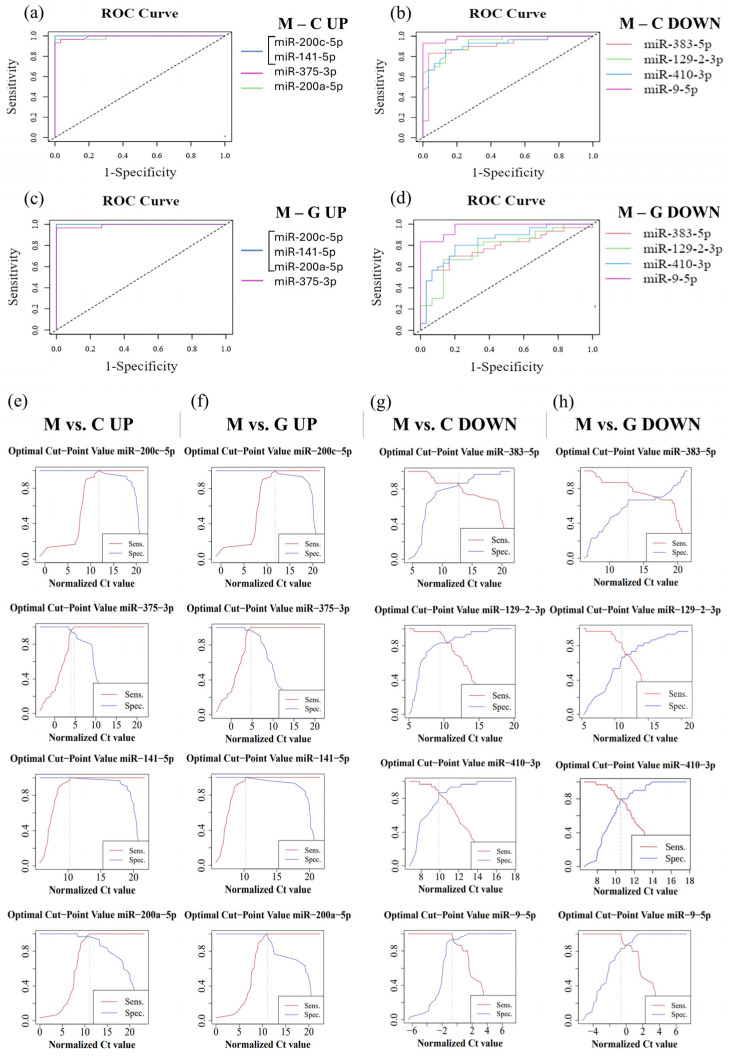
ROC analysis performed on normalized data from LUAD-BM patients, controls, and GBM patients to determine the efficacy of individual miRNAs. (**a**) ROC curves of upregulated hsa-miR-200c-5p (AUC: 1), hsa-miR-141-5p (AUC: 1), hsa-miR-375-3p (AUC: 0.993), and hsa-miR-200a-5p (AUC: 0.99) obtained from the LUAD-BM and control comparison; (**b**) ROC curves of downregulated hsa-miR-383-5p (AUC: 0.934), hsa-miR-129-2-3p (AUC: 0.907), hsa-miR-410-3p (AUC: 0.916), and hsa-miR-9-5p (AUC: 0.989) obtained from the LUAD-BM and control comparison; (**c**) ROC curves of upregulated hsa-miR-200c-5p (AUC: 1), hsa-miR-141-5p (AUC: 1), hsa-miR-375-3p (AUC: 0.991), and hsa-miR-200a-5p (AUC: 1) obtained from the LUAD-BM and GBM comparison; (**d**) ROC curves of downregulated hsa-miR-383-5p (AUC: 0.791), hsa-miR-129-2-3p (AUC: 0.776), hsa-miR-410-3p (AUC: 0.838), and hsa-miR-9-5p (AUC: 0.971) obtained from the LUAD-BM and GBM comparison; (**e**) determination of the optimal cut-point values of hsa-miR-200c-5p (optimal cut-point: 13.05), hsa-miR-141-5p (optimal cut-point: 17.92), hsa-miR-375-3p (optimal cut-point: 4.19, 5.31), and hsa-miR-200a-5p (optimal cut-point: 13.18) in the comparison of LUAD-BM and control samples; (**f**) determination of the optimal cut-point values of hsa-miR-200c-5p (optimal cut-point: 12.46), hsa-miR-141-5p (optimal cut-point: 13.29), hsa-miR-375-3p (optimal cut-point: 5.88), and hsa-miR-200a-5p (optimal cut-point: 11.12) in the comparison of LUAD-BM and control samples; (**g**) determination of the optimal cut-point values of hsa-miR-383-5p (optimal cut-point: 12.52), hsa-miR-129-2-3p (optimal cut-point: 8.5), hsa-miR-410-3p (optimal cut-point: 9.87), and hsa-miR-9-5p (optimal cut-point: −1.37) in the comparison of LUAD-BM and GBM samples; (**h**) determination of the optimal cut-point values of hsa-miR-383-5p (optimal cut-point: 12.72), hsa-miR-129-2-3p (optimal cut-point: 10.99), hsa-miR-410-3p (optimal cut-point: 10.43), and hsa-miR-9-5p (optimal cut-point: −0.75) in the comparison of LUAD-BM and control samples.

In order to evaluate the diagnostic sensitivity and specificity of individual miRNAs, we performed ROC analysis using normalized Ct values obtained from RT-qPCR. During the analysis, the normalized data of LUAD-BM patients were compared with those of control and GBM groups (Table 2). For the LUAD-BM and control comparison, ROC curves (Figure 10a) showed that upregulated hsa-miR-200c-5p and hsa-miR-141-5p exhibited perfect diagnostic performance, with an AUC of 1, a sensitivity of 1, and a specificity of 1 (Figure 10e). The AUC of upregulated hsa-miR-375-3p was 0.993, with sensitivity and specificity values of 0.967 (Figure 10a,e). For upregulated hsa-miR-200a-5p, the AUC was 0.99, with a sensitivity of 1 and a specificity of 0.967 (Figure 10e). For downregulated miRNAs, the AUC value for hsa-miR-383-5p was 0.934, with sensitivity and specificity values of 0.867; hsa-miR-129-2-3p displayed an AUC of 0.907, with sensitivity of 0.967 and specificity of 0.833; hsa-miR-410-3p had an AUC of 0.916, with sensitivity and specificity values of 0.867; while the AUC for hsa-miR-9-5p was 0.989, with a sensitivity of 1 and specificity of 0.933, as depicted in Figure 10b,g. For the LUAD-BM and GBM comparison, the upregulated hsa-miR-200c-5p, hsa-miR-141-5p, and hsa-miR-200a-5p showed an AUC of 1, with both sensitivity and specificity values at 1, and hsa-miR-375-3p had an AUC of 0.991, with a sensitivity of 1 and a specificity of 0.967, as shown in Figure 10c,f. Among the downregulated miRNAs, the AUC values (Figure 10d) were 0.791, 0.776, 0.838, and 0.971 for hsa-miR-383-5p (sensitivity: 0.867; specificity: 0.7), hsa-miR-129-2-3p (sensitivity: 0.833; specificity: 0.7), hsa-miR-410-3p (sensitivity: 0.8; specificity: 0.8), and hsa-miR-9-5p (sensitivity: 1; specificity: 0.833), respectively (Figure 10h). The results indicate that hsa-miR-200c-5p, hsa-miR-141-5p, hsa-miR-375-3p, hsa-miR-200a-5p, hsa-miR-383-5p, hsa-miR-129-2-3p, hsa-miR-410-3p, and hsa-miR-9-5p may serve as effective diagnostic markers for LUAD-BM and for distinguishing it from GBM based on the sensitivity and specificity values obtained.

**Figure 11:**
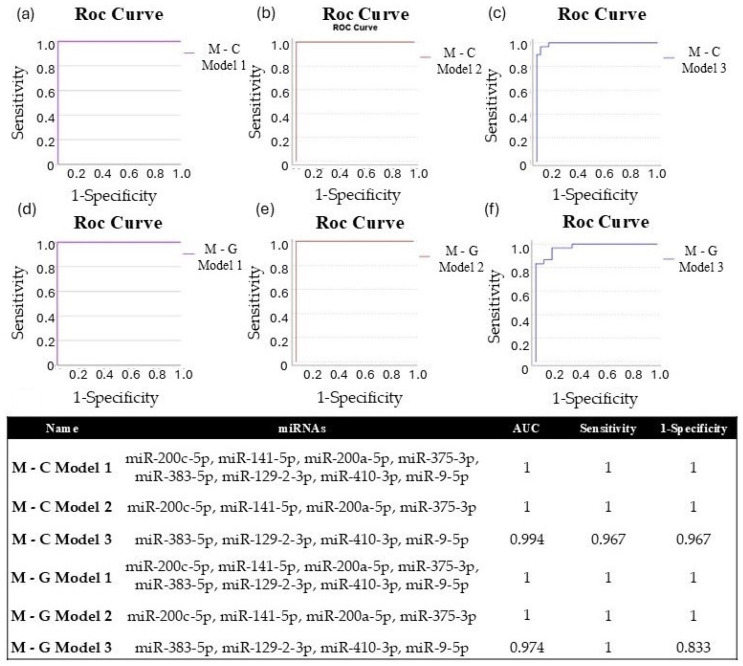
ROC curves of (**a**) M-C Model 1 (miR-200c-5p, miR-141-5p, miR-200a-5p, miR-375-3p, miR-383-5p, miR-129-2-3p, miR-410-3p, miR-9-5p), (**b**) M-C Model 2 (miR-200c-5p, miR-141-5p, miR-200a-5p, miR-375-3p), and (**c**) M-C Model 3 (miR-383-5p, miR-129-2-3p, miR-410-3p, miR-9-5p) created to differentiate LUAD-BM patients and controls; ROC curves of (**d**) M-G Model 1 (miR-200c-5p, miR-141-5p, miR-200a-5p, miR-375-3p, miR-383-5p, miR-129-2-3p, miR-410-3p, miR-9-5p), (**e**) M-G Model 2 (miR-200c-5p, miR-141-5p, miR-200a-5p, miR-375-3p), and (**f**) M-G Model 3 (miR-383-5p, miR-129-2-3p, miR-410-3p, miR-9-5p) were created to differentiate LUAD-BM and GBM patients; the composition of miRNA diagnostic models was developed using the binary logistic regression method, highlighting the AUC, sensitivity, and specificity values achieved.

We applied binary logistic regression to develop miRNA diagnostic models, enabling us to assess the diagnostic potential of combined miRNAs. For both experimental comparisons (metastasis–control; metastasis–GBM), we evaluated the diagnostic efficiency of eight significantly different miRNAs (Model 1: miR-200c-5p, miR-141-5p, miR-200a-5p, miR-375-3p, miR-383-5p, miR-129-2-3p, miR-410-3p, and miR-9-5p). Additionally, we conducted the same analysis using the four upregulated (Model 2: miR-200c-5p, miR-141-5p, miR-200a-5p, and miR-375-3p) and four downregulated (Model 3: miR-383-5p, miR-129-2-3p, miR-410-3p, and miR-9-5p) miRNAs for both study groups. For each model, a Y score greater than 0.5 was classified as LUAD-BM, while a Y score less than 0.5 indicated normal or GBM. The diagnostic specificity and sensitivity of the models are assessed through the ROC curves, which are demonstrated in Figure 11a–f. For both M-C and M-G, the AUC value was 1 for Models 1 and 2, with sensitivity and specificity values of 1, and the AUC was 0.994 for M-C Model 3 (sensitivity: 0.967; specificity: 0.967) and 0.974 for M-G Model 3 (sensitivity: 1; specificity: 0.833). These data suggest that although individual miRNAs may be effective in diagnosing LUAD-BM and differentiating it from GBM, the combined models presented higher sensitivity and specificity.

**Figure 12:**
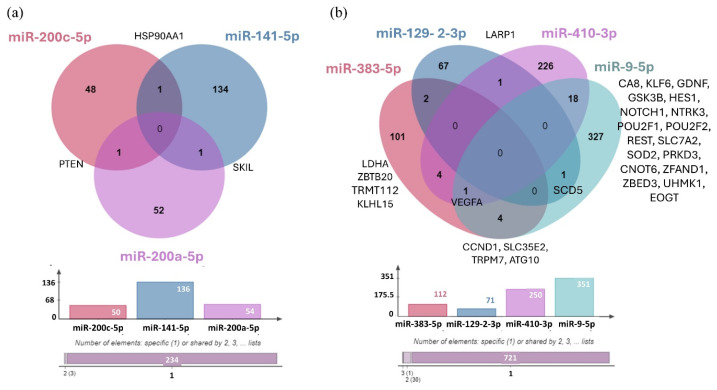
(**a**) Venn Diagram illustrating the distribution of target genes for the three upregulated miRNAs validated by RT-qPCR. (**b**) The distribution of target genes for the four downregulated miRNAs validated by RT-qPCR. The bar chart represents the number of validated targets identified in miRTarBase 9.0: hsa-miR-200c-5p (50 targets), hsa-miR-141-5p (136 targets), hsa-miR-200a-5p (54 targets), hsa-miR-383-5p (112 targets), hsa-miR-129-2-3p (71 targets), hsa-miR-410-3p (250 targets), and hsa-miR-9-5p (351 targets). No target genes were identified for hsa-miR-375-3p.

Using the miRTarBase 9.0 database, we identified the validated target genes of three upregulated miRNAs (hsa-miR-200c-5p, hsa-miR-141-5p, and hsa-miR-200a-5p) and four downregulated miRNAs (hsa-miR-383-5p, hsa-miR-129-2-3p, hsa-miR-410-3p, and hsa-miR-9-5p) (Figure 12). We were unable to identify any validated target genes for hsa-miR-375-3p.

## 4. Discussion

The incidence of BM exceeds that of primary nervous system tumors, approximately 16% of which originate from LUAD [28]. After the development of metastasis, the patient’s prognosis is extremely poor, with an average survival of ~2 months without treatment (~12 months with treatment) [29]. Among primary tumors, GBM is the most common malignant brain tumor, with a median survival of about 4–6 months without treatment (~14 months with treatment), that belongs to the group of grade IV gliomas according to the WHO classification [30]. The differentiation of BM and GBM is based on MRI imaging and histopathological analysis. However, due to the presence of similar hyperintense and necrotic areas in both BM and GBM, conventional MRI often results in similar appearances for these lesions, limiting its effectiveness in distinguishing between them [14]. Therapy of both tumor types differs significantly, and early diagnosis could significantly improve the effectiveness of oncotherapy.

### 4.1. Identification of Differentially Expressed miRNAs and Pathway Enrichment Analysis

KEGG pathway analysis demonstrated that our significantly upregulated miRNAs are implicated in a number of tumor processes, including regulation of the cell cycle and apoptosis, as well as focal adhesion and tight junction biology and regulation of adherens junction-related proteins, p53, Wnt, TGF-beta, and the ErbB signaling pathway (Figure 7). Dysregulation of the p53 protein is implicated in the development and metastasis of many cancers. Due to its tumor suppressor properties, p53 plays a key role in cell cycle regulation and cell growth, as well as in DNA repair mechanisms [31,32]. As a transcription factor, it regulates the expression of several target genes that encode anti-angiogenic and pro-apoptotic proteins. In addition, several studies have reported that p53 can modulate autophagic processes, contributing to the fine-tuning of cell death [33]. Thus, p53 deregulation may contribute to tumor development and invasion in several ways, including its reduced function, leading to uncontrolled cell division and genetic instability while inhibiting apoptosis and autophagy, which promote cancer cell survival, especially under adverse conditions such as hypoxia or nutrient deprivation. Downregulation of adherens junction and tight junction proteins such as claudin, occludin, and E-cadherin disrupts cell adhesion and promotes the migration of tumor cells, thereby playing a key role in tumor metastasis [34,35]. Li et al. showed that Enolase 1 (ENO1) can enhance cell migration in lung cancer by regulating the Hepatocyte growth factor receptor (HGFR) and Wnt signaling pathway-driven epithelial–mesenchymal transition (EMT) [36]. EMT plays a fundamental role in the migration of tumor cells and their acquisition of invasive capacity. It is also known that the main inducer of EMT is the TGF-β, which cooperates with pathways such as Wnt, Ras, and Notch [37,38,39,40,41].

GO biological process analysis results further showed that these significantly downregulated miRNAs primarily target genes that play key roles in processes such as regulation of apoptosis, cell proliferation, programmed cell death, and cell cycle, while downregulated miRNAs were linked to the positive regulation of cell proliferation (Figure 8).

### 4.2. Validation of Differentially Expressed miRNAs by RT-qPCR

The differential expression of has-miR-200c-5p has been found to be associated with colorectal cancer and hepatocellular carcinoma [42,43]. However, a comprehensive review of the extant literature failed to identify any evidence that other research groups have investigated the expression of this particular microRNA in lung tumors or lung cancer-related BM. Previously, Chen et al. reported that circ-Zinc finger E-box-binding homeobox 1 (circ-ZEB1) sponges inhibit the function of miR-200c-5p and promote tumor progression, EMT, and chemotherapy resistance in colorectal cancer cells [44]. Furthermore, overexpression of ADAM Metallopeptidase with Thrombospondin Type 1 Motif 5 (ADAMTS5) is associated with poor prognosis in lung cancer patients, and Liu and Yao et al. proved that ADAMTS5 is targeted by miR-200c-5p [45,46]. Contrary to our results, this study suggests that reduced expression of microRNA-200c-5p is associated with the development of metastasis. However, the data presented here suggest that elevated levels of miR-200c-5p expression may play a role in the development of metastases in patients with lung adenocarcinoma.

Wu et al. identified the upregulation of hsa-miR-141-5p in lung lesions, highlighting its potential as a biomarker and therapeutic target [47]. Moreover, similar to our results, Roskova et al. reported differential expression of miR-141-5p in lung cancer BM [48].

High expression levels of miR-200a-5p have been associated with several malignancies, including LUAD, breast cancer, papillary thyroid carcinoma, and colon cancer [49,50,51,52]. Recently, Bilski et al. have demonstrated that increased expression of miRNAs such as miR-200a-3p and miR-141-3p is associated with higher overall survival (OS) in patients with WHO grade II/III brain gliomas, while decreased expression of miR-200a-5p, miR-200c-5p, and miR-429 is associated with worse OS at both two and five years [53].

In the case of hsa-miR-375-3p, it was proposed that the downregulation of Tyrosine 3-Monooxygenase/Tryptophan 5-Monooxygenase Activation Protein Zeta (YWHAZ) by this miRNA may serve to suppress cell migration and invasion in NSCLC [54]. Conversely, Mao et al. demonstrated that exosomal hsa-miR-375-3p, secreted by small-cell lung cancer (SCLC), may facilitate metastasis by disrupting endothelial barriers by targeting claudin-1 [55]. However, limited information is available on hsa-miR-375-3p, and no validated target genes have been identified thus far in databases. This highlights the importance of further exploring this miRNA’s potential role in tumor regulation.

It has been shown that miR-383-5p is downregulated in LUAD, and its expression level is correlated with tumor size, differentiation, and patient survival. Upregulation of hsa-miR-383-5p has been reported to inhibit LUAD progression by decreasing Cellular Inhibitor Of PP2A (CIP2A) protein levels [56]. The tumor suppressor role of hsa-miR-383-5p was additionally confirmed by Xiaoqian et al., demonstrating its ability to suppress LUAD development under physiological conditions via inhibition of TMPO Antisense RNA 1 (TMPO-AS1) [57]. Another study identified that resveratrol inhibits cell proliferation, migration, and invasion by regulating the Metastasis-associated Lung Adenocarcinoma Transcript 1 (MALAT1)/miR-383-5p/DNA-damage-inducible transcript 4 (DDIT4) pathway that finally induces apoptosis [58]. These findings suggest that the downregulation of hsa-miR-383-5p may play a role in metastasis development. Our findings are consistent with these results and provide further evidence supporting its potential involvement in LUAD-BM progression.

Focusing on miR-410-3p, Li et al. demonstrated that a high level of miR-410-3p inhibited the proliferation, migration, and invasion of glioma cells through the inhibition of the transforming growth factor-ß receptor type 2 (TGFBR2) gene under Propofol treatment [59]. Our analysis revealed significantly decreased expression levels of miR-410-3p in LUAD-BM samples compared to both control and GBM groups; however, no significant differences were observed between GBM and control groups.

Several studies have highlighted the tumor suppressor function of miR-129-3p under physiological conditions. Chen et al. showed that reduced mir-129-2-3p expression suppresses the proliferation and invasion of intrahepatic cholangiocarcinoma through its target gene Protein Phosphatase, Mg2+/Mn2+-Dependent 1D (Wip1) [60]. Tao et al. reported that miR-129-2-3p has an inhibitory effect on proliferation, motility, and tumor growth in colon cancer cells and also reported significantly reduced levels of miR-129-2-3p in colon cancer tissue [61]. Consistent with these findings, we observed reduced expression of miR-129-2-3p in LUAD-BM samples compared to controls.

Several studies have reported that high expression of miR-9-5p contributes to tumor cell migration. Zhu et al. demonstrated that highly expressed miR-9-5p facilitates LUAD cell migration and invasion by modulating the Inhibitor Of DNA Binding 4 (ID4) [62]. However, in our study, we observed reduced miR-9-5p expression in LUAD-BM samples compared to both GBM and control groups. Another study reported that highly expressed miR-9-5p is associated with a better prognosis in GBM and inhibits glioma cell proliferation through suppression of forkhead box P2 (FOXP2) [63]. In contrast, our study found no significant differences in miR-9-5p expression between GBM patients and controls. However, we found reduced miR-9-5p expression in LUAD-BM samples related to GBM and control samples. The observed discrepancies between studies may be attributed to genetic and expression level variations in microRNAs among different racial and ethnic groups [64].

It is generally accepted that the altered expression of a single miRNA is not sufficiently specific to reliably identify a particular tumor type. Additionally, it has been reported that simultaneous determination of the expression of multiple miRNAs increases their diagnostic accuracy and reliability [65]. Related to that, we analyzed the combined AUC, sensitivity, and specificity values of three miRNA panel models (Model 1: miR-200c-5p, miR-141-5p, miR-200a-5p, miR-375-3p, miR-383-5p, miR-129-2-3p, miR-410-3p, and miR-9-5p; Model 2: miR-200c-5p, miR-141-5p, miR-200a-5p, and miR-375-3p; Model 3: miR-383-5p, miR-129-2-3p, miR-410-3p, and miR-9-5p) in LUAD-BM–control and LUAD-BM–GBM comparisons For both comparisons, the AUC value was 1 for Model 1 and Model 2, with sensitivity and specificity values of 1. In the case of Model 3 in LUAD-BM–control comparison, the AUC value increased to 0.994 with a sensitivity and specificity of 96.7%. Meanwhile, the AUC value of Model 3 in the LUAD-BM–GBM comparison was 0.974 (sensitivity: 100%; specificity: 83.3%). Our findings highlight the potential benefits of combining miRNAs from both prognostic and diagnostic perspectives.

The common target gene shared by hsa-miR-200c-5p and hsa-miR-141-5p is Heat Shock Protein 90 Alpha Family Class A Member 1 (HSP90AA1), while SKI Like Proto-Oncogene (SKIL) is the shared target of hsa-miR-141-5p and hsa-miR-200a-5p, and PTEN is the common target of hsa-miR-200c-5p and hsa-miR-200a-5p. Studies have shown that genetic variations within genes of the PI3K–PTEN–AKT–mTOR signaling pathway can serve as predictive markers for the risk of BM in patients with NSCLC [66]. In the case of downregulated miRNAs, it was observed that hsa-miR-129-2-3p and hsa-miR-410-3p regulate the La-related protein 1 (LARP1) gene, while Stearoyl-CoA Desaturase 5 (SCD5) is a shared target of hsa-miR-129-2-3p and hsa-miR-9-5p. Additionally, Cyclin D1 (CCND1), Solute Carrier Family 35 Member E2B (SLC35E2), Transient Receptor Potential Cation Channel Subfamily M Member 7 (TRPM7), and Autophagy-related 10 (ATG10) were identified as common target genes of hsa-miR-383-5p and hsa-miR-9-5p. As shown previously, overexpression of CCND1 has been associated with the development and progression of various tumor types, including NSCLC [67,68]. Furthermore, aberrant TRPM7 expression is associated with poor lung cancer prognosis, with its overexpression playing a critical role in tumor cell migration and invasion [69,70]. In addition, the downregulated hsa-mir-383-5p and hsa-mir-410-3p microRNAs shared four common target genes, including Lactate dehydrogenase A (LDHA), Zinc Finger and BTB Domain Containing 20 (ZBTB20), TRNA Methyltransferase Activator Subunit 11-2 (TRMT112), and KLHL15. On the other hand, the hsa-miR-410-3p and hsa-miR-9-5p miRNAs share the highest number of experimentally validated target genes, including Carbonic Anhydrase 8 (CA8), KLF Transcription Factor 6 (KLF6), Glial Cell-derived Neurotrophic Factor (GDNF), Glycogen Synthase Kinase 3 Beta (GSK3B), Hes Family BHLH Transcription Factor 1 (HES1), Notch Receptor 1 (NOTCH1), Neurotrophic Receptor Tyrosine Kinase 3 (NTRK3), POU Class 2 Homeobox 1 (POU2F1), POU Class 2 Homeobox 2 (POU2F2), RE1 Silencing Transcription Factor (REST), Solute Carrier Family 7 Member 2 (SLC7A2), Superoxide Dismutase 2 (SOD2), Protein Kinase D3 (PRKD3), CCR4-NOT Transcription Complex Subunit 6 (CNOT6), Zinc Finger AN1-Type Containing 1 (ZFAND1), Zinc Finger BED-Type Containing 3 (ZBED3), U2AF Homology Motif Kinase 1 (UHMK1), and EGF Domain Specific O-Linked N-Acetylglucosamine Transferase 9 (EOGT). Deregulation of GDNF has been linked to mechanisms driving glioma proliferation, migration, and invasion, while REST plays a critical role in CNS development, and its deregulation has been associated with several disorders like axon guidance errors in differentiating neurons, impaired neuronal formation, neurodevelopmental disorders, and brain tumors [71,72]. We also identified Vascular Endothelial Growth Factor A (VEGFA) as a common target gene regulated by three downregulated miRNAs: hsa-miR-383-5p, hsa-miR-410-3p, and hsa-miR-9-5p.

To our knowledge, other research groups have identified miR-9-5p, miR-21-5p, miR-184-5p, miR-197-5p, miR-328-5p, miR-330-3p, miR-375-5p, miR-378-5p, miR-423-5p, let-7a, miR-145-5p, miR-199a-5p, miR-217-5p, miR-596-3p, and miR-1207-5p with BMs from NSCLC [73]. This high degree of heterogeneity in miRNA expression can be attributed to population-specific genetic variations, which can influence the baseline expression of these miRNAs. These variations result in differences in miRNA expression levels across racial and ethnic groups, as well as between geographic regions [64,74,75]. Therefore, we consider it important to establish a geographically and ethnically specific miRNA panel to facilitate the differentiation of LUAD-BM from the control group and GBM patients. Geographical and ethnic affiliation may be regarded as a crucial factor for the clinical applicability of miRNA biomarkers.

To the best of our knowledge, this is the first study that demonstrated the deregulation of hsa-miR-200c-5p, hsa-miR-200a-5p, hsa-miR-383-5p, and hsa-miR-410-3p in LUAD-BM compared to both control and GBM samples. Our report is the first to demonstrate the co-deregulation of hsa-miR-200c-5p, hsa-miR-141-5p, hsa-miR-200a-5p, hsa-miR-375-3p, hsa-miR-383-5p, hsa-miR-129-2-3p, hsa-miR-410-3p, and hsa-miR-9-5p expression in LUAD-BM compared to both control and GBM samples, as well.

## 5. Conclusions

The identification of differentially expressed miRNAs holds enormous potential for enhancing the diagnosis and prognosis of various tumors. A panel of eight miRNAs, including hsa-miR-200c-5p, hsa-miR-141-5p, hsa-miR-200a-5p, hsa-miR-375-3p, hsa-miR-383-5p, hsa-miR-129-2-3p, hsa-miR-410-3p, and hsa-miR-9-5p was determined using NGS and RT-qPCR, which can discriminate LUAD-BM, GBM, and control brain tissue samples with outstanding sensitivity and specificity individually. In combination, these miRNAs have the potential to be used to diagnose LUAD-BM and to distinguish it from GBM in the Hungarian population with 100% sensitivity and specificity. Our functional annotation analysis revealed that the differentially expressed miRNAs identified by NGS are key regulators of tumorigenesis and metastasis, which confirms the pathophysiological role of miRNAs in various tumor processes. While the small sample size is a limitation of this study, we recommend further validation of these findings on a larger cohort to strengthen the clinical applicability of this miRNA panel.

## Figures and Tables

**Table 1:** Summary of the characteristics of the 6 GBM, 6 LUAD-BM, and 6 control patients selected for NGS analysis.

Characreristic	Gender	Age	Immunohistochemical Characteristics
LUAD-BM_1	M	67	CK7 +, TTF-1+, CD×2 −
LUAD-BM_2	M	71	CK7 +, TTF-1+, CD×2 −
LUAD-BM_3	M	73	CK7 +, TTF-1+, CD×2 −
LUAD-BM_4	F	66	CK7 +, TTF-1+, CD×2 −
LUAD-BM_5	F	71	CK7 +, TTF-1+, CD×2 −
LUAD-BM_6	F	59	CK7 +, TTF-1+, CD×2 −
GBM_1	M	60	IDH wild type
GBM_2	M	56	IDH wild type
GBM_3	M	65	IDH wild type
GBM_4	F	69	IDH wild type
GBM_5	F	57	IDH wild type
GBM_6	F	70	IDH wild type
Controll_1	M	70	-
Controll_2	M	52	-
Controll_3	M	52	-
Controll_4	F	71	-
Controll_5	F	80	-
Controll_6	F	61	-

**Table 2:** Significance analysis and ROC analysis of individual miRNAs based on normalized Ct values obtained from RT-qPCR, comparing LUAD-BM patient data to those of control and GBM patients.

Differentially Expressed miRNAs		UP				DOWN			
		miR-200c-5p	miR-141-5p	miR-375-3p	miR-200a-5p	miR-383-5p	miR-129-2-3p	miR-410-3p	miR-9-5p
Mann-Whitney U test	M ↔ C	<0.00001	<0.00001	<0.00001	<0.00001	<0.00001	<0.00001	<0.00001	<0.00001
AUC		1	1	0.993	0.99	0.934	0.907	0.916	0.989
Optimal Cut-Off Point		13.05	17.92	4.19 5.31	13.18	12.52	8.5	9.87	−1.37
Sensitivity		1	1	0.967	1	0.867	0.967	0.867	1
Specificity		1	1	0.967	0.967	0.867	0.833	0.867	0.933
Mann-Whitney U test	M ↔ G	<0.00001	<0.00001	<0.00001	<0.00001	<0.00001	0.00026	<0.00001	<0.00001
AUC		1	1	0.991	1	0.791	0.776	0.838	0.971
Optimal Cut-Off Point		12.46	13.29	5.88	11.12	12.72	10.99	10.43	−0.75
Sensitivity		1	1	1	1	0.867	0.833	0.8	1
Specificity		1	1	0.967	1	0.7	0.7	0.8	0.833

## Data Availability

The LUAD-BM and GBM RNA-seq data underlying this article are available in NCBI Gene Expression Omnibus (GEO) database at https://www.ncbi.nlm.nih.gov/geo/ and can be accessed with GSE284777. For the control samples, we used a dataset originally associated with another unpublished article at the time of submission. These data are available in the Gene Expression Omnibus (GEO) database (https://www.ncbi.nlm.nih.gov/geo/ under the accession number GSE244332.

## Supplementary Materials

The following supporting information can be downloaded at: https://www.mdpi.com/article/10.3390/cancers17040581/s1, Table S1: List of the 229 significantly deregulated miRNAs in tissue samples of LUAD-BM comparing with their expression in control group, listed according to their expression levels. Among them, 118 miRNAs were upregulated while 111 miRNAs were downregulated. Table S2: List of the 46 significantly deregulated miRNAs in tissue samples of LUAD-BM compared with their expression in GBM group, listed according to their expression levels. Among them, 30 miRNAs were upregulated while 16 miRNAs were downregulated. Table S3: List of the 15 most strongly downregulated and upregulated miRNAs in tissue samples of LUAD-BM compared with their expression in the control and GBM group, listed according to their expression levels.
