# Supplementary material for: Construction of a miRNA Panel for Differentiating Lung Adenocarcinoma Brain Metastases and Glioblastoma
Source: Cancers (Basel). 2025 Feb 8;17(4):581. doi: 10.3390/cancers17040581 (PMC11853152; doi:10.3390/cancers17040581)
Supplement: Supplementary file 1 [file cancers-17-00581-s001.zip › Table S1.pdf]

Table S1. List of the 229 significantly deregulated miRNAs in tissue samples of LUAD-BM comparing with their expression in Control group, listed according to their expression levels. Among them 118 miRNAs were upregulated while 111 miRNAs were downregulated.

**BM-LUAD – Control** (UP)			
**Regulation**	**miRNA**	**logFC**	**adj-P-Val**
**UP**	HSA-MIR-200C-3P	9.236660023	3.65E-06
**UP**	HSA-MIR-375-3P	8.917612387	0.000556309
**UP**	HSA-MIR-21-3P	7.880115707	8.81E-05
**UP**	HSA-MIR-200A-5P	7.64026087	2.92E-05
**UP**	HSA-MIR-210-3P	7.372334767	8.81E-05
**UP**	HSA-MIR-141-3P	7.266670477	1.53E-06
**UP**	HSA-MIR-200A-3P	7.262304313	4.99E-05
**UP**	HSA-MIR-10A-5P	7.14916651	0.000864793
**UP**	HSA-MIR-141-5P	6.988811017	1.53E-06
**UP**	HSA-MIR-200B-3P	6.462992733	0.000176013
**UP**	HSA-MIR-429	6.11923412	0.000136921
**UP**	HSA-MIR-214-3P	5.471112253	0.002182505
**UP**	HSA-MIR-182-5P	5.456388003	0.00114866
**UP**	HSA-MIR-96-5P	5.363271117	0.002222947
**UP**	HSA-MIR-452-5P	5.180669915	0.008812729
**UP**	HSA-MIR-147B-3P	4.991828427	0.002134568
**UP**	HSA-MIR-21-5P	4.799467967	0.00102287
**UP**	HSA-MIR-148A-5P	4.640099333	0.006581381
**UP**	HSA-MIR-27A-5P	4.59136857	0.000864793
**UP**	HSA-MIR-130B-3P	4.56850095	0.000694819
**UP**	HSA-MIR-200B-5P	4.552542093	0.000609721
**UP**	HSA-MIR-576-3P	4.512213725	0.000864793
**UP**	HSA-MIR-214-5P	4.457307649	0.009143627
**UP**	HSA-MIR-1266-5P	4.317679777	4.99E-05
**UP**	HSA-MIR-183-5P	4.274650367	0.003014693
**UP**	HSA-MIR-19B-3P	4.27427616	0.000493402
**UP**	HSA-MIR-17-3P	4.236468177	0.005510772
**UP**	HSA-MIR-200C-5P	4.158788717	0.00115944
**UP**	HSA-MIR-146B-5P	4.141913633	0.003331234
**UP**	HSA-MIR-130A-5P	4.00685542	0.015805705
**UP**	HSA-MIR-210-5P	3.892706127	0.005255699
**UP**	HSA-MIR-503-5P	3.881168772	0.000694819
**UP**	HSA-MIR-10B-5P	3.84279863	0.005056548
**UP**	HSA-MIR-199B-5P	3.823788847	0.001937291
**UP**	HSA-MIR-146B-3P	3.69141957	0.007730533
**UP**	HSA-MIR-671-5P	3.60600298	0.030288257

**UP**	HSA-MIR-199A-5P	3.600735727	0.006581381
**UP**	HSA-MIR-196B-5P	3.537931967	0.008812729
**UP**	HSA-MIR-18A-3P	3.484697967	0.004358794
**UP**	HSA-MIR-130A-3P	3.481217603	0.024714774
**UP**	HSA-MIR-193A-3P	3.329298732	0.008999351
**UP**	HSA-MIR-146A-5P	3.327944033	0.008409772
**UP**	HSA-MIR-28-3P	3.3240413	0.000704754
**UP**	HSA-MIR-217-5P	3.294969031	0.018569275
**UP**	HSA-LET-7G-3P	3.268452423	0.009143627
**UP**	HSA-MIR-29A-5P	3.196190774	0.01056158
**UP**	HSA-MIR-25-5P	3.076384117	0.001641439
**UP**	HSA-MIR-152-5P	3.052566855	0.001641439
**UP**	HSA-MIR-6510-3P	3.046231517	0.038542302
**UP**	HSA-MIR-19A-3P	3.045505593	0.002540307
**UP**	HSA-MIR-34A-5P	2.991419287	0.000556309
**UP**	HSA-MIR-629-5P	2.990596607	0.004329693
**UP**	HSA-MIR-92A-1-5P	2.94027276	0.01251271
**UP**	HSA-MIR-7974	2.873277505	0.019601199
**UP**	HSA-MIR-3934-5P	2.835756267	0.001719874
**UP**	HSA-MIR-551A	2.828831933	0.029150583
**UP**	HSA-MIR-425-5P	2.759169697	0.008812729
**UP**	HSA-MIR-365A-5P	2.74598309	0.00953049
**UP**	HSA-MIR-16-1-3P	2.730569917	0.00131795
**UP**	HSA-MIR-532-3P	2.705852778	0.036670601
**UP**	HSA-MIR-155-5P	2.671169697	0.014949873
**UP**	HSA-MIR-222-3P	2.661845403	0.036716487
**UP**	HSA-MIR-27A-3P	2.641385457	0.000175742
**UP**	HSA-MIR-29B-3P	2.6203379	0.02411381
**UP**	HSA-MIR-92B-5P	2.611345245	0.030255635
**UP**	HSA-MIR-148A-3P	2.588536267	0.048887691
**UP**	HSA-MIR-193A-5P	2.52171865	0.00911739
**UP**	HSA-MIR-193B-3P	2.505837243	0.009143627
**UP**	HSA-MIR-574-3P	2.49276584	0.003406148
**UP**	HSA-MIR-19B-1-5P	2.46635921	0.010522418
**UP**	HSA-MIR-106B-5P	2.441687517	0.005540324
**UP**	HSA-MIR-378A-3P	2.411961633	0.008812729
**UP**	HSA-MIR-1307-5P	2.39173269	0.000442605
**UP**	HSA-MIR-542-3P	2.391408233	0.045669513
**UP**	HSA-MIR-1304-3P	2.368232861	0.000210294
**UP**	HSA-MIR-23A-3P	2.35986828	0.00114866
**UP**	HSA-MIR-339-5P	2.346736783	0.009764519

**UP**	HSA-MIR-3158-3P	2.326236223	0.025808226
**UP**	HSA-MIR-362-3P	2.32590141	0.038020371
**UP**	HSA-MIR-493-3P	2.324577893	0.027912004
**UP**	HSA-MIR-378A-5P	2.264352547	0.011221565
**UP**	HSA-MIR-22-3P	2.262929533	0.008999351
**UP**	HSA-MIR-580-3P	2.232460409	8.14E-05
**UP**	HSA-MIR-505-3P	2.231182017	0.039781135
**UP**	HSA-MIR-4677-3P	2.191528167	0.00359363
**UP**	HSA-MIR-376B-3P	2.149775003	0.049505881
**UP**	HSA-MIR-186-5P	2.131227967	0.000610139
**UP**	HSA-MIR-1307-3P	2.114028317	0.002246597
**UP**	HSA-MIR-337-5P	2.108369983	0.000864793
**UP**	HSA-MIR-424-5P	2.107859633	0.034224828
**UP**	HSA-MIR-10399-5P	2.067253733	0.013657696
**UP**	HSA-MIR-4326	2.04759606	0.042655829
**UP**	HSA-LET-7I-3P	2.032722627	0.00545666
**UP**	HSA-MIR-142-3P	2.03172625	0.030288257
**UP**	HSA-MIR-548E-3P	1.9813464	0.002036376
**UP**	HSA-MIR-93-3P	1.960667077	0.00911739
**UP**	HSA-MIR-125A-3P	1.957534198	0.015186242
**UP**	HSA-MIR-378I	1.913434314	0.032070481
**UP**	HSA-MIR-26B-3P	1.828567633	0.000525746
**UP**	HSA-MIR-3913-5P	1.788795825	0.017680604
**UP**	HSA-MIR-574-5P	1.76133466	0.038542302
**UP**	HSA-MIR-24-3P	1.73405011	0.000981414
**UP**	HSA-MIR-324-5P	1.71783737	0.033224973
**UP**	HSA-MIR-652-3P	1.672133967	0.027793559
**UP**	HSA-MIR-223-3P	1.658850213	0.02411381
**UP**	HSA-MIR-3679-5P	1.59510198	0.00115944
**UP**	HSA-MIR-25-3P	1.570215133	0.008949067
**UP**	HSA-MIR-422A	1.554836955	0.027403134
**UP**	HSA-MIR-34A-3P	1.51915549	0.046982595
**UP**	HSA-MIR-151A-3P	1.503980433	0.033100541
**UP**	HSA-MIR-151A-5P	1.47987577	0.02411381
**UP**	HSA-MIR-4470	1.450520083	0.011949205
**UP**	HSA-MIR-152-3P	1.4335093	0.006874491
**UP**	HSA-MIR-423-5P	1.329758567	0.007730533
**UP**	HSA-MIR-7705	1.318997415	0.025808226
**UP**	HSA-MIR-627-5P	1.29050025	0.038316213
**UP**	HSA-MIR-24-2-5P	1.226608622	0.045669513
**UP**	HSA-MIR-345-5P	1.032724007	0.032430766

**BM-LUAD – Control** (DOWN)			
**Regulation**	**miRNA**	**logFC**	**adj-P-Val**
**DOWN**	HSA-MIR-135A-5P	-5.677099155	0.00102287
**DOWN**	HSA-MIR-323A-3P	-5.57043264	0.002134568
**DOWN**	HSA-MIR-656-3P	-5.459175432	0.000704754
**DOWN**	HSA-MIR-433-3P	-5.40984825	0.00131795
**DOWN**	HSA-MIR-383-5P	-5.3643089	0.00102287
**DOWN**	HSA-MIR-129-5P	-5.356577717	0.004358794
**DOWN**	HSA-MIR-129-1-3P	-5.300697463	0.000864793
**DOWN**	HSA-MIR-9-3P	-5.200237133	0.00142972
**DOWN**	HSA-MIR-411-5P	-5.118635333	0.001445604
**DOWN**	HSA-MIR-889-3P	-5.1087528	0.005035464
**DOWN**	HSA-MIR-539-3P	-5.07898869	0.002134568
**DOWN**	HSA-MIR-124-3P	-4.993781955	0.00102287
**DOWN**	HSA-MIR-490-3P	-4.97197547	0.007673945
**DOWN**	HSA-MIR-129-2-3P	-4.925791283	0.005035464
**DOWN**	HSA-MIR-885-5P	-4.87559715	0.000556309
**DOWN**	HSA-MIR-128-3P	-4.78856755	0.000400634
**DOWN**	HSA-MIR-410-3P	-4.73009819	0.001038116
**DOWN**	HSA-MIR-9-5P	-4.641059417	0.000694819
**DOWN**	HSA-MIR-153-3P	-4.63807718	0.002540307
**DOWN**	HSA-MIR-1197	-4.59204851	0.003163322
**DOWN**	HSA-MIR-139-5P	-4.528608833	0.002540307
**DOWN**	HSA-MIR-124-5P	-4.495739055	0.000842647
**DOWN**	HSA-MIR-543	-4.264113293	0.003024773
**DOWN**	HSA-MIR-628-5P	-4.250571483	3.49E-05
**DOWN**	HSA-MIR-144-5P	-4.184598127	0.01733055
**DOWN**	HSA-MIR-598-3P	-4.116230073	0.00142972
**DOWN**	HSA-MIR-329-3P	-4.092149117	0.000864793
**DOWN**	HSA-MIR-204-5P	-4.032331383	0.005476797
**DOWN**	HSA-MIR-219A-2-3P	-4.026441783	0.027552583
**DOWN**	HSA-MIR-499A-5P	-3.923567333	0.002747236
**DOWN**	HSA-MIR-369-3P	-3.808176873	0.001719874
**DOWN**	HSA-MIR-485-3P	-3.772271413	0.001641439
**DOWN**	HSA-MIR-218-5P	-3.72422015	0.00142972
**DOWN**	HSA-MIR-95-3P	-3.640190923	0.010218107
**DOWN**	HSA-MIR-139-3P	-3.638678467	0.022096004
**DOWN**	HSA-MIR-340-3P	-3.63410946	0.001395812
**DOWN**	HSA-MIR-495-3P	-3.6263224	0.000441252
**DOWN**	HSA-MIR-1-3P	-3.61337572	0.00318162
**DOWN**	HSA-MIR-592	-3.574869177	0.005201727

**DOWN**	HSA-MIR-487B-3P	-3.537598083	0.00142972
**DOWN**	HSA-MIR-409-5P	-3.53189585	0.006600615
**DOWN**	HSA-MIR-539-5P	-3.481070982	0.00142972
**DOWN**	HSA-MIR-655-3P	-3.471859407	0.001479195
**DOWN**	HSA-MIR-204-3P	-3.39126573	0.00142972
**DOWN**	HSA-MIR-1224-3P	-3.310660307	0.008812729
**DOWN**	HSA-MIR-330-5P	-3.264481797	0.007730533
**DOWN**	HSA-MIR-340-5P	-3.2176065	0.001719874
**DOWN**	HSA-MIR-487A-3P	-3.180631437	0.00820955
**DOWN**	HSA-MIR-370-3P	-3.11383495	0.006087571
**DOWN**	HSA-MIR-4705	-3.05259725	0.002540307
**DOWN**	HSA-MIR-154-3P	-2.968265933	0.002036376
**DOWN**	HSA-MIR-346	-2.939926153	0.022003077
**DOWN**	HSA-MIR-758-3P	-2.909672317	0.000400634
**DOWN**	HSA-MIR-382-3P	-2.900627573	0.00115944
**DOWN**	HSA-MIR-125B-2-3P	-2.872820563	0.006874491
**DOWN**	HSA-MIR-1185-1-3P	-2.868153963	0.016317227
**DOWN**	HSA-MIR-374B-3P	-2.864030063	0.016674744
**DOWN**	HSA-MIR-132-5P	-2.82305503	0.050716259
**DOWN**	HSA-MIR-412-5P	-2.807369965	0.016108014
**DOWN**	HSA-MIR-3912-3P	-2.800203345	0.000685291
**DOWN**	HSA-MIR-301A-5P	-2.787718405	0.005225632
**DOWN**	HSA-MIR-136-3P	-2.777393317	0.005169651
**DOWN**	HSA-MIR-421	-2.768843487	0.006600615
**DOWN**	HSA-MIR-431-3P	-2.754329322	0.005346272
**DOWN**	HSA-MIR-6868-3P	-2.744870797	0.018913765
**DOWN**	HSA-MIR-363-3P	-2.737095683	0.009764519
**DOWN**	HSA-MIR-379-5P	-2.697472933	0.000864793
**DOWN**	HSA-MIR-767-5P	-2.663371893	0.010696201
**DOWN**	HSA-MIR-149-5P	-2.615756877	0.015186242
**DOWN**	HSA-MIR-1296-5P	-2.555100673	0.000864793
**DOWN**	HSA-MIR-3085-3P	-2.41586685	0.010038674
**DOWN**	HSA-MIR-3200-3P	-2.36182364	0.036716487
**DOWN**	HSA-MIR-1249-3P	-2.295990405	0.035599537
**DOWN**	HSA-MIR-381-3P	-2.290371667	0.032070481
**DOWN**	HSA-MIR-7-5P	-2.288056767	0.011949205
**DOWN**	HSA-MIR-105-5P	-2.27776187	0.002747236
**DOWN**	HSA-MIR-195-5P	-2.255198033	0.031485116
**DOWN**	HSA-MIR-487A-5P	-2.239112463	0.012224073
**DOWN**	HSA-MIR-20B-5P	-2.222400673	0.033342795
**DOWN**	HSA-MIR-299-5P	-2.189116	0.035204957

**DOWN**	HSA-MIR-330-3P	-2.168574693	0.006087571
**DOWN**	HSA-MIR-138-2-3P	-2.098848948	0.009143627
**DOWN**	HSA-LET-7E-3P	-2.04441845	0.046590319
**DOWN**	HSA-MIR-382-5P	-2.038298283	0.010730818
**DOWN**	HSA-MIR-885-3P	-2.005152677	0.010897065
**DOWN**	HSA-MIR-107	-1.988707633	0.009143627
**DOWN**	HSA-MIR-328-3P	-1.937035017	0.008949067
**DOWN**	HSA-MIR-140-5P	-1.92526321	0.003046042
**DOWN**	HSA-MIR-3943	-1.917662357	0.02411381
**DOWN**	HSA-MIR-433-5P	-1.854953892	0.00114866
**DOWN**	HSA-MIR-6516-3P	-1.831326898	0.039748838
**DOWN**	HSA-MIR-1271-5P	-1.785292953	0.013657696
**DOWN**	HSA-MIR-3117-3P	-1.689891913	0.040568802
**DOWN**	HSA-MIR-1185-5P	-1.684777007	0.042655829
**DOWN**	HSA-MIR-181D-5P	-1.661703017	0.006855697
**DOWN**	HSA-MIR-369-5P	-1.607546086	0.018320537
**DOWN**	HSA-MIR-30C-5P	-1.605568467	0.01259573
**DOWN**	HSA-MIR-154-5P	-1.602639447	0.014089268
**DOWN**	HSA-MIR-125B-5P	-1.5959694	0.006874491
**DOWN**	HSA-MIR-708-5P	-1.58323079	0.037534323
**DOWN**	HSA-MIR-1185-2-3P	-1.565525928	0.031697013
**DOWN**	HSA-MIR-26A-5P	-1.511784633	0.007730533
**DOWN**	HSA-MIR-494-3P	-1.500415877	0.008227351
**DOWN**	HSA-MIR-488-5P	-1.44039635	0.015805705
**DOWN**	HSA-LET-7E-5P	-1.3966183	0.045669513
**DOWN**	HSA-LET-7D-3P	-1.391808933	0.00114866
**DOWN**	HSA-MIR-1179	-1.391284207	0.003331234
**DOWN**	HSA-LET-7A-5P	-1.37193175	0.030593293
**DOWN**	HSA-MIR-6866-5P	-1.359483232	0.01323489
**DOWN**	HSA-LET-7G-5P	-1.2223313	0.035599537
**DOWN**	HSA-MIR-744-5P	-1.054788577	0.027552583
