# Supplementary material for: Construction of a miRNA Panel for Differentiating Lung Adenocarcinoma Brain Metastases and Glioblastoma
Source: Cancers (Basel). 2025 Feb 8;17(4):581. doi: 10.3390/cancers17040581 (PMC11853152; doi:10.3390/cancers17040581)
Supplement: Supplementary file 1 [file cancers-17-00581-s001.zip › Table S2.pdf]

Table S2. List of the 46 significantly deregulated miRNAs in tissue samples of LUAD-BM comparing with their expression in Control group, listed according to their expression levels. Among them 30 miRNAs were upregulated while 16 miRNAs were downregulated.

**BM-LUAD – GBM (UP)**			
**Regulation**	**miRNA**	**logFC**	**adj-P-Val**
**UP**	hsa-mir-375-3P	9.026995017	4.79E-04
**UP**	hsa-mir-200C-3P	8.4249083	2.48409E-06
**UP**	hsa-mir-141-5P	6.988811017	1.41E-07
**UP**	hsa-mir-141-3P	6.874607983	1.51E-05
**UP**	hsa-mir-200A-5P	6.05133965	6.95E-06
**UP**	hsa-mir-200B-3P	5.544456133	1.63E-04
**UP**	hsa-mir-200A-3P	5.543739967	1.63E-04
**UP**	hsa-mir-200B-5P	4.605332433	0.000375602
**UP**	hsa-mir-429	4.543697433	4.79E-04
**UP**	hsa-mir-147B-3P	4.308940533	0.012344664
**UP**	hsa-mir-200C-5P	4.158788717	0.001154457
**UP**	hsa-mir-210-3P	3.70224185	0.019060022
**UP**	hsa-mir-1266-5P	3.68464235	0.00064818
**UP**	hsa-mir-21-3P	3.488940967	0.021184197
**UP**	hsa-mir-338-3P	3.350775333	0.00246845
**UP**	hsa-mir-874-3P	3.036463367	0.02184562
**UP**	hsa-mir-326	3.014962483	0.037166563
**UP**	hsa-mir-92A-1-5P	2.9930631	0.02150492
**UP**	hsa-mir-3934-5P	2.835756267	0.002107923
**UP**	hsa-mir-1307-3P	2.7172002	0.00820212
**UP**	hsa-mir-130B-3P	2.64603625	0.043267713
**UP**	hsa-mir-25-5P	2.595049867	0.020087693
**UP**	hsa-mir-425-5P	2.5079332	0.023512124
**UP**	hsa-mir-1307-5P	2.440671517	1.15E-03
**UP**	hsa-mir-19B-3P	2.414687033	0.037166563
**UP**	hsa-mir-580-3P	2.239353383	0.000163111
**UP**	hsa-mir-29C-5P	1.965278917	0.025660271
**UP**	hsa-mir-28-3P	1.896772167	0.043990037
**UP**	hsa-mir-1304-3P	1.8096787	0.024501662
**UP**	hsa-mir-29C-3P	1.800112067	0.011573987

BM-LUAD – GBM (DOWN)			
**Regulation**	**miRNA**	**logFC**	**adj-P-Val**
**DOWN**	hsa-mir-135A-5P	-5.702480752	0.006986303
**DOWN**	hsa-mir-9-3P	-5.6283381	0.001383942
**DOWN**	hsa-mir-9-5P	-4.593640917	0.00064818
**DOWN**	hsa-mir-204-5P	-4.237767883	0.031510738
**DOWN**	hsa-mir-10B-3P	-3.532786547	0.016680473
**DOWN**	hsa-mir-125B-2-3P	-3.4623158	0.003823452
**DOWN**	hsa-mir-195-5P	-3.422598183	0.009590525
**DOWN**	hsa-mir-92B-3P	-3.395942433	0.000737287
**DOWN**	hsa-mir-450A-5P	-3.20995944	0.025660271
**DOWN**	hsa-mir-195-3P	-3.119985417	0.032289831
**DOWN**	hsa-mir-421	-2.789920317	0.015186748
**DOWN**	hsa-mir-1185-1-3P	-2.766632157	0.043999669
**DOWN**	hsa-mir-181D-5P	-2.48497355	0.006586611
**DOWN**	hsa-mir-708-5P	-2.245933617	0.011573987
**DOWN**	hsa-mir-125B-5P	-1.897585833	0.008362825
**DOWN**	hsa-mir-140-5P	-1.553081567	0.025092541
