# Supplementary material for: Construction of a miRNA Panel for Differentiating Lung Adenocarcinoma Brain Metastases and Glioblastoma
Source: Cancers (Basel). 2025 Feb 8;17(4):581. doi: 10.3390/cancers17040581 (PMC11853152; doi:10.3390/cancers17040581)
Supplement: Supplementary file 1 [file cancers-17-00581-s001.zip › Table S3.pdf]

Table S3. List of the 15 most strongly down-regulated and up-regulated miRNAs in tissue samples of BM-LUAD patients, compared with their expression in the control group and GBM patients, ranked according to their expression levels.

	BM-LUAD – Control		BM-LUAD – GBM	
**Regulation**	**miRNA**	**logFC**	**miRNA**	**logFC**
**UP**	hsa-miR-200c-3P	9.24	hsa-miR-375-3P	9.03
**UP**	hsa-miR-375-3P	8.92	hsa-miR-200c-3P	8.42
**UP**	hsa-miR-21-3P	7.88	hsa-miR-141-5P	6.99
**UP**	hsa-miR-200a-5P	7.64	hsa-miR-141-3P	6.87
**UP**	hsa-miR-210-3P	7.37	hsa-miR-200a-5P	6.05
**UP**	hsa-miR-141-3P	7.27	hsa-miR-200b-3P	5.54
**UP**	hsa-miR-200a-3P	7.26	hsa-miR-200a-3P	5.54
**UP**	hsa-miR-10a-5P	7.15	hsa-miR-200b-5P	4.61
**UP**	hsa-miR-141-5P	6.99	hsa-miR-429	4.54
**UP**	hsa-miR-200b-3P	6.46	hsa-miR-147b-3P	4.31
**UP**	hsa-miR-429	6.12	hsa-miR-200c-5P	4.16
**UP**	hsa-miR-214-3P	5.47	hsa-miR-210-3P	3.70
**UP**	hsa-miR-182-5P	5.46	hsa-miR-1266-5P	3.68
**UP**	hsa-miR-96-5P	5.36	hsa-miR-21-3P	3.49
**UP**	hsa-miR-452-5P	5.18	hsa-miR-338-3P	3.35
**DOWN**	hsa-miR-135a-5P	-5.68	hsa-miR-135a-5P	-5.70
**DOWN**	hsa-miR-323a-3P	-5.57	hsa-miR-9-3P	-5.63
**DOWN**	hsa-miR-656-3P	-5.46	hsa-miR-9-5P	-4.59
**DOWN**	hsa-miR-433-3P	-5.41	hsa-miR-204-5P	-4.24
**DOWN**	hsa-miR-383-5P	-5.36	hsa-miR-10b-3P	-3.53
**DOWN**	hsa-miR-129-5P	-5.36	hsa-miR-125b-2-3P	-3.46
**DOWN**	hsa-miR-129-1-3P	-5.30	hsa-miR-195-5P	-3.42
**DOWN**	hsa-miR-9-3P	-5.20	hsa-miR-92b-3P	-3.40
**DOWN**	hsa-miR-411-5P	-5.12	hsa-miR-450a-5P	-3.21
**DOWN**	hsa-miR-889-3P	-5.11	hsa-miR-195-3P	-3.12
**DOWN**	hsa-miR-539-3P	-5.08	hsa-miR-421	-2.79
**DOWN**	hsa-miR-124-3P	-4.99	hsa-miR-1185-1-3P	-2.77
**DOWN**	hsa-miR-490-3P	-4.97	hsa-miR-181d-5P	-2.48
**DOWN**	hsa-miR-129-2-3P	-4.93	hsa-miR-708-5P	-2.25
**DOWN**	hsa-miR-885-5P	-4.88	hsa-miR-125b-5P	-1.90
